# Association of aortic wall thickness with stiffness in the Multi-Ethnic Study of Atherosclerosis (MESA)

**DOI:** 10.1186/1532-429X-16-S1-P83

**Published:** 2014-01-16

**Authors:** Chia-Ying Liu, Doris Chen, David Bluemke, Colin Wu, Gisela Teixido-Tura, Atul Chugh, Sujethra Vasu, Gregory Hundley, Joao A Lima

**Affiliations:** 1 Radiology and Imaging Sciences, National Institutes of Health, Bethesda, Maryland, USA; 2 Department of Radiology, Johns Hopkins Hospital, Baltimore, Maryland, USA; 3 Office of Biostatistics Research, National Institutes of Health, Bethesda, Maryland, USA; 4 Division of cardiovascular medicine, University of Louisville School of Medicine, Louisville, Maryland, USA; 5 Deparment of Internal Medicine, Wake Forest University, Winston-Salem, North Carolina, USA; 6 Department of Cardiology, Hospital General Universitari Vall dHebron, Barcelona, Spain

## Background

The coupling of aortic wall thickening and stiffening has been recognized in hypertension. However, specific relationship between aortic wall thickness (AWT) and stiffness has not yet been documented in community-based studies. The purpose of this study is to evaluate associations between AWT and arterial stiffness measured by aortic distensibility and PWV in the MESA cohort.

## Methods

423 studies by 1.5-T whole-body MRI were analyzed. CMRI protocol included cardiac function and late gadolinium enhancement. Only participants with no findings of myocardial scar were included. AWT images were obtained using a double inversion recovery black-blood fast spin-echo sequence and phase contrast cine gradient echo sequence was used to evaluate aortic stiffness. Aortic sagittal oblique plane with black blood sequence was acquired to position the aortic imaging and allowed for the measurement of the distance between the ascending and descending aorta. Images of the ascending and descending aorta were obtained in the transverse plane at the level of the right pulmonary artery perpendicular to the vessel lumen. The thickness of the midthoracic descending aortic wall was measured using electronic calipers at 4 standard positions: 12, 3, 6, and 9 o'clock (QMASS 7.2). The average value of these 4 measurements was calculated. Distensibiliy of the ascending aorta and PWV were performed using validated automated software (ARTFUN. INSERM U678).

## Results

Table 1 lists demographics, AWT, distensibility and PWV stratified by hypertension status. AWT was not different (p = 0.35) but distensibility was lower (p < 0.001) and PWV was higher (p = 0.012) in hypertension. Linear regression analyses (Table 2) demonstrate distensibility was significantly correlated to AWT in the cohort without hypertension. AWT was a predictor for PWV with the basic adjustment (Model 1) in the hypertension group, but this correlation diminished after adjusting for more variables (Model 2, only systolic blood pressure and black race were positively correlated to PWV significantly).

**Table 1:** Mean characteristics of the MESA participants stratified by hypertension status

mean ± SD	With hypertension (N = 264)	W/O hypertension (N = 159)	p-value
Age (years)	72 ± 8.5	69 ± 8.6	< 0.001

Race (% of white/black)	51/49	77/23	< 0.001

Body mass index (kg/m2)	29.3 ± 5.3	27.7 ± 5.1	0.002

Men (%)	38	49	0.03

Systolic blood pressure (mmHg)	131 ± 22	114 ± 13	< 0.001

Diastolic blood pressure (mmHg)	69 ± 11	66 ± 10	0.012

Metabolic Syndrome, (%)*	45	17	< 0.001

LV End diastolic volume (ml)	120 ± 32	120 ± 29	0.99

LV End systolic volume (ml)	46 ± 20	48 ± 15	0.34

LV End diastolic mass (g)	129 ± 37	119 ± 32	0.006

LV Stroke volume (ml)	74 ± 18	72 ± 18	0.36

LV Ejection Fraction (%)	62 ± 7.6	61 ± 6.5	0.007

Aortic wall thickness (mm)	2.68 ± 0.27	2.66 ± 0.26	0.35

Distensibility of ascending aorta (%/mmhg)	0.18 ± 0.11	0.25 ± 0.18	< 0.001

Pulse wave velocity (m/s)	9.5 ± 4.4	8.5 ± 2.8	0.012

* Metabolic Syndrome was defined by NCEP guidelines (Circ 2004;109;433-438)

**Table 2:** Regression models for association of distensibility and PWV with AWT (Regression coefficients B/P).

		With hypertension (N = 264)		W/O hypertension (N = 159)	
		
		B	P	B	P
Distensibility of ascending aorta (%/mmhg)	Model 1	-0.017	0.54	0.16	0.003
	
	Model 2	-0.016	0.54	0.164	0.001

Pulse wave velocity (m/s)	Model 1	2.32	0.046	0.52	0.6
	
	Model 2	1.7	0.144	0.65	0.5

Model 1: multivariable analysis accounting for age, sex, race, and BMI. Model 2: adjusted for variables in model 1 in addition to systolic blood pressure, diabetes mellitus, LDL, HDL, current smoking status, and smoking pack years.

## Conclusions

We have demonstrated aortic functional alterations with hypertension (lower distensibility and higher PWV) and the coupling between vessel wall thickness and function. Without hypertension, morphological changes is associated more strongly with distensibility, however, PWV are more affected by aortic wall thickening in hypertension.

## Funding

This research was supported by contracts N01-HC-95159 through N01-HC-95168 from the National Heart, Lung, and Blood Institute.

